# Augmented Oscillations in QT Interval Duration Predict Mortality Post Myocardial Infarction Independent of Heart Rate

**DOI:** 10.3389/fphys.2020.578173

**Published:** 2020-11-09

**Authors:** Fatima J. El-Hamad, Safa Y. Bonabi, Alexander Müller, Alexander Steger, Georg Schmidt, Mathias Baumert

**Affiliations:** ^1^School of Electrical and Electronic Engineering, The University of Adelaide, Adelaide, SA, Australia; ^2^School of Electronic and Telecommunications Engineering, RMIT University, Melbourne, VIC, Australia; ^3^Internal Medicine I Department, Technical University of Munich, Munich, Germany

**Keywords:** repolarization variability, risk stratification, sudden death, myocardial infarction, autoregressive model, cardiovascular disease

## Abstract

**Objective:**

This study seeks to decompose QT variability (QTV) into physiological sources and assess their role for risk stratification in patients post myocardial infarction (MI). We hypothesize that the magnitude of QTV that cannot be explained by heart rate or respiration carries important prognostic information.

**Background:**

Elevated beat-to-beat QTV is predictive of cardiac mortality, but the underlying mechanisms, and hence its interpretation, remain opaque.

**Methods:**

We decomposed the QTV of 895 patients post MI into contributions by heart rate, respiration, and unexplained sources.

**Results:**

Cox proportional hazard analysis demonstrates that augmented oscillations in QTV and their level of dissociation from heart rate are associated with a higher 5-year mortality rate (18.4% vs. 4.7%, *p* < 0.0001). In patients with left ventricular ejection fraction (LVEF) > 35%, a higher QTV risk score was associated with a significantly higher 5-year mortality rate (16% vs. 4%, *p* < 0.0001). In patients with a GRACE score ≥ 120, a higher QTV risk score was associated with a significantly higher 5-year mortality (25% vs. 11%, *p* < 0.001).

**Conclusion:**

Augmented oscillations in QTV and discordance from heart rate, possibly indicative of excessive sympathetic outflow to the ventricular myocardium, predict high risk in patients post MI independent from established risk markers.

**Clinical Trial Registration:**

www.ClinicalTrials.gov, identifier NCT00196274.

## Introduction

Myocardial infarction (MI) is the most common cause of sudden cardiac death (SCD); over 80% of fatal arrhythmias are caused by structural coronary arterial abnormalities and their consequences (Huikuri et al., 2001). Prediction of SCD currently poses a clinical challenge and identification of high-risk patients needs to be considerably improved (Myerburg, 2001).

Autonomic dysfunction and electrical instability are key factors predisposing MI patients to ventricular tachycardia and ventricular fibrillation and consequent SCD (Schwartz et al., 1992; Liew, 2010; Wellens et al., 2014). Indices of ventricular repolarization lability, such as QT variability (QTV), have attracted considerable interest in the area of risk stratification as they allow for a non-invasive investigation of the autonomic nervous system influence on the ventricular myocardium and the lability of the ventricular repolarization process (Myerburg, 2001; Wellens et al., 2014). Measures of QTV (QTVI, SDQT) are increased post MI and their prognostic value has been demonstrated in several studies (Wellens et al., 2014; Baumert et al., 2016a). QTV was increased in six out of the 12-lead ECG recording of patients with recent MI even after covarying for low T wave amplitude (Hasan et al., 2013) and in post MI patients with reduced LVEF compared to those with preserved LVEF and those with uncomplicated coronary heart disease (Sosnowski et al., 2002). QTV was also increased in coronary disease patients with old MI when compared to those without MI (Yao et al., 2019). QTVI has been shown to be predictive of SCD and all-cause mortality in patients with LVEF between 35% and 40% (Piccirillo et al., 2007). In 24-h Holter recordings of patients with acute MI, QT/RR variability ratio was found to be predictive of all-cause mortality independent of other established risk markers (Jensen et al., 2005).

Beat-to-beat fluctuations in the QT interval (representing the variability of the repolarization process) are the result of a complex process that involves multiple physiological mechanisms. The rate adaptation of the QT interval constitutes a large fraction of QTV in the normal heart (El-Hamad et al., 2015; Baumert et al., 2016b). Respiration also influences QTV indirectly through respiratory sinus arrhythmia (Sutherland et al., 1983; Baumert et al., 2016b). Furthermore, ventricular repolarization is directly influenced by the autonomic nervous system (Merri et al., 1993) via the dense innervation of sympathetic nerves into the ventricular myocardium, contributing to rate-independent QTV (Hall, 2011). Hence, in the compromised heart, excessive sympathetic outflow might initiate lethal ventricular arrhythmias (Ng, 2016).

In the context of MI, few studies report on the rate-independent component of repolarization variability. Zhu et al. (2008) found that repolarization variability independent of heart rate was increased in MI patients compared to age-matched healthy subjects, while the rate-dependent component was not different between the two groups. Others found no difference in the percentage of RR-dependent RT interval variability between MI patients and age-matched healthy subjects (Lombardi et al., 1998). Comparing MI patients with reduced LVEF to those with preserved LVEF and to another group with coronary artery disease but no MI, Sosnowski et al. (2002) reported an increase in overall RT variability and specifically an increase in its HF power, suggesting an important role for respiration. They also observed an uncorrelation between RR and RT interval variability in MI patients with depressed LVEF compared to the other two groups.

There are only few reports on QTV’s individual components in patients post MI, and none on their individual role in risk stratification post MI. In this study, we hypothesize that the component of QTV that cannot be explained by heart rate or respiration, possibly reflecting sympathetic influences on the ventricles, carries important prognostic information. To test this hypothesis, we decomposed QTV into three components (hart rate dependent, respiratory dependent, and independent component) and assessed their individual roles in risk stratification in patients post MI.

## Materials and Methods

### Study Cohort

We analyzed high-resolution ECG recorded previously for cardiac risk stratification post MI (Barthel et al., 2013). A total of 941 MI survivors were enrolled between March 2000 and May 2005. Acute MI was diagnosed based on at least two of the following findings: typical chest pain lasting ≥ 20 min, creatine kinase above twice the upper normal limit of the respective laboratory, and admission ST-segment elevation ≥ 0.1 mV in at least two contiguous limb leads or ≥ 0.2 mV in at least two contiguous precordial leads (Bernard et al., 1979). Eligible patients had survived acute MI less than four weeks before recruitment, they were aged 80 years or less, had sinus rhythm, and did not meet the criteria for secondary prophylactic implantation of implantable cardioverter defibrillator before hospital discharge. A total of 32 patients did not pass the initial screening due to atrial fibrillation (Sinnecker et al., 2015).

The main outcome measure was total mortality during a follow-up period of 5 years (once every 6 months), where the last follow-up was performed in May 2010. The study was conducted at the hospital of the Technische Universität München, the German Heart Centre, and the Klinikum Rechts der Isar, both in Munich, Germany. The study protocol was approved by the local ethics committee, and written consent from patients was obtained.

### Measurements

ECG (Porti System, TMS, Netherlands) and thoracic respiratory signals (piezoelectric thoracic sensor; Pro-Tech, United States) were recorded at 1.6 kHz for 30 min in each patient within 2 weeks of index MI. Patients were studied in the morning in the supine position without interruption of their normal medication regimen. The GRACE score, including the age of the patient, a history of past heart failure, a history of past MI, serum creatinine at admission, the cardiac biomarker status at admission, systolic blood pressure at admission, the pulse at admission, ST-deviation at admission, and in-hospital percutaneous coronary intervention, was selected for predicting the long-term prognosis (Eagle et al., 2004). Details of other recorded signals can be found elsewhere (Barthel et al., 2013).

### Data Pre-processing

All ECG signals were visually inspected for signal quality; recordings with poorly defined T wave were excluded from the analysis based on subjective assessment. Beat-to-beat heart period (RR) and beat-to-beat Q peak to T end interval (QT) were obtained from all ECGs. We used an automated template-based algorithm that tracks QT changes beat by beat with high accuracy and robust to noise (Schmidt et al., 2014). The QT interval was automatically determined on the template beat using a slope method and manually adjusted if necessary.

The thoracic respiratory signal was sampled at the occurrence of the R-peak in the ECG signal to obtain beat-to-beat values of respiration. All beat-to-beat series (RR, QT, respiration) were visually inspected to select stationary segments of around 350 consecutive beats. All selected RR segments were then visually validated against the ECG to identify QRS detection errors and irregular heart rhythms. Recordings with RR segments consisting of more than 10% ventricular or supraventricular ectopic beats (EBs) were excluded from the analysis, while ectopic beats were mathematically interpolated in recordings with less than 10% EBs using the spline interpolation method. QT, RR and respiration time series were detrended using the smoothness priors method (Tarvainen et al., 2002) comprising a time-varying finite-impulse response high-pass filter with a corner frequency of 0.04Hz. The detrended data segments were normalized to zero mean and unit variance for power spectral analysis.

### Model-Based QTV Analysis

Power contribution analysis was performed to decompose QTV into heart period dependent, respiratory dependent and unexplained contributions.

We employed a linear autoregressive model with two external inputs (ARXX) to decompose QT variability (Baselli et al., 1997). We chose an open-loop structure, where the rhythm sources generating the three signals are assumed uncorrelated, which allows the decomposition of QTV power into contributions by the different inputs. Thus, the autoregressive model was defined as:

(1) $$A_{1}\left(z\right)QT\left(i\right)=B_{1}\left(z\right)RR\left(i\right)+B_{2}\left(z\right)Resp\left(i\right)+e_{QT}\left(i\right)$$

Equation (1) describes QTV as a function of its own past, past and present values of heart period (RR) and respiration (Resp) and a noise source that represents actual noise and rhythms originating from sources not accounted for in the model. Heart period was modeled as autoregressive model with respiration as an external input, while respiration was modeled as a separate autoregressive process. Details of the mathematical models and their parameters are described elsewhere (Baselli et al., 1997; El-Hamad et al., 2015).

The Akaike information criterion (Akaike, 1974) was used to select the model order of the multivariate autoregressive process from the range 6 to 12. Model parameters were estimated using the least squares method. Resulting models were validated by assessing the correlation between model residuals and whiteness of model noise sources (Porta et al., 1998; El-Hamad et al., 2015).

The following variables were computed:

• QT_*mean*_–average QT interval (in ms).
• QT_*c*_–rate-corrected average QT interval, using Bazett’s formula (in ms).
• QTV_*tota*__*l*_–variance of beat-to-beat QT intervals (in ms^2^).
• QTV_*respiration*_–variance of beat-to-beat QT intervals related to respiration (in %).
• QTV_*RR*_–variance of beat-to-beat QT intervals related to heart period (in %).
• QTV_*unexplained*_–variance of beat-to-beat QT intervals independent from heart period and respiration (in %).

### Statistics

Univariate Cox regression analysis was performed on each demographic variable to assess its predictive value for mortality; expressed as hazard ratios (HR) with 95% confidence intervals (CI). To test whether the characteristics of patients who were excluded from analysis were significantly different from those in the final study cohort, we compared both groups using the two-sample Wilcoxon test and the chi-squared test for continuous and categorical variables, respectively.

The predictive value of the different QT variability measures was assessed using univariate Cox regression analysis. The significant predictors resulting from the univariate analysis were then included in a multivariable Cox regression analysis with the stepAIC method (Hothorn and Lausen, 2003). The linear predictor model resulting from the multivariate cox regression analysis is termed ‘QTV risk score’ which includes QTV measures which were found to contribute independently to risk prediction. The optimal cut-off for the QTV risk score was calculated by the Maximally Selected Rank Statistics (Hothorn and Lausen, 2003; Lausen et al., 2004). We chose the maximum of the log-rank statistics with at least 90% of the observations in group 1 and no more than 90% of the observations in group 1 as constraints. To assess the additive predictive value of the QTV risk score beyond established clinical risk markers, we performed multivariable Cox regression analysis using the QTV risk score as well as a model consisting of the following clinical predictor variables: GRACE score, LVEF, presence of diabetes, chronic obstructive pulmonary disease and expiration-triggered respiratory sinus arrhythmia. Kaplan–Meier survival curves were computed for the QTV risk score and for subgroup analysis based on LVEF ≤ 35% and GRACE ≥ 120 points. A *p*-value < 0.05 was considered significant. All statistics were performed with R (R Core Team, 2014).

## Results

### Data Pre-processing Results

Out of 941 recordings, we excluded 20 from the analysis due to poor signal quality. After manually selecting stationary segments of QT, RR and respiration, another 26 recordings were excluded due to the high percentage of irregular heartbeats (more than 10% ventricular or supraventricular ectopic beats; or bigeminus rhythm). Of the 895 patients included in the analysis, 62 died during the follow-up period. Table 1 shows the clinical characteristics and demographics for subjects included in the final analysis compared to those excluded from the analysis. Mortality was higher in the group of excluded patients (10 deaths). On average, those patients were significantly older, had a higher GRACE score and had a lower estimated glomerular filtration rate (Table 1).

**TABLE 1:** Patient demographics and hazard ratios of the entire study population as well as the comparison of characteristics of included versus excluded patients.

Variables	Study population (*n* = 941)	Hazard Ratio* (99% CI)	*p*-value	Included patients (*n* = 895)	Excluded patients (*n* = 46)	Included versus excluded (*p*-value)
Age (years), median (IQR)	60.9 (51.6–68.8)	1.09 (1.06–1.12)	<0.001	60.7 (51.5–68.6)	66.6 (58.9–73.1)	0.004
Females, *n* (%)	182 (19.3)	1.22 (0.67–2.21)	0.52	173 (19.3)	9 (19.6)	1.00
Diabetes mellitus, *n* (%)	184 (19.6)	2.72 (1.63–4.53)	<0.001	176 (19.7)	8 (17.4)	0.85
History of previous MI, *n* (%)	90 (9.6)	3.34 (1.87–5.97)	<0.001	84 (9.4)	6 (13)	0.57
Hypertension, *n* (%)	682 (72.5)	1.58 (0.84–2.96)	0.16	652 (72.8)	30 (65.2)	0.34
Smoking, *n* (%)	488 (51.9)	0.88 (0.54–1.45)	0.62	461 (51.5)	27 (58.7)	0.42
COPD, *n* (%)	39 (4.1)	3.85 (1.83–8.08)	<0.001	37 (4.1)	2 (4.3)	1
CK max (U/l), median (IQR)	1302 (646–2460)	1 (1–1)	0.89	1316 (648–2475)	1106 (599–2040)	0.64
LVEF (%), median (IQR)	53 (45–60)	0.95 (0.94–0.97)	<0.001	53 (45–60)	51.5 (38–58.75)	0.23
MI localization						
Anterior, *n* (%)	391 (41.5)	1.08 (0.65–1.78)	0.77	375 (41.9)	16 (34.8)	0.42
Posterior, *n* (%)	435 (46.2)	0.79 (0.47–1.31)	0.36	409 (45.7)	26 (56.5)	0.2
Lateral, *n* (%)	102 (10.8)	1.62 (0.83–3.18)	0.17	98 (10.9)	4 (8.7)	0.81
Unclassified, *n* (%)	12 (1.3)	0 (0–Inf)	0.996	12 (1.3)	0 (0 0)	0.91
BMI (kg/m^2^), median (IQR)	26.6 (24.5–29.1)	1.02 (0.96–1.09)	0.52	26.6 (24.5–29.0)	25.1 (24.1–28.7)	0.26
Serum creatinine (mg/dL), median (IQR)	1.1 (0.9–1.3)	1.77 (1.46–2.15)	<0.001	1.1 (0.9–1.3)	1.1 (1–1.3)	0.29
Cardiogenic shock/CPR, *n* (%)	41 (4.4)	0.81 (0.2–3.32)	0.77	36 (4)	5 (10.9)	0.07
Intervention						
PCI, *n* (%)	878 (93.3)	0.65 (0.28–1.51)	0.31	835 (93.3)	43 (93.5)	1
Thrombolysis, *n* (%)	14 (1.5)	0 (0–Inf)	0.996	14 (1.6)	0 (0 0)	0.82
CABG, *n* (%)	6 (0.6)	3.36 (0.47–24.22)	0.23	5 (0.6)	1 (2.2)	0.7
No revascularization possible, *n* (%)	43 (4.6)	1.91 (0.77–4.77)	0.16	41 (4.6)	2 (4.3)	1
Aspirin, *n* (%)	913 (97)	0.56 (0.18–1.78)	0.33	869 (97.1)	44 (95.7)	0.91
Clopidogrel, *n* (%)	920 (97.8)	0.4 (0.13–1.29)	0.13	876 (97.9)	44 (95.7)	0.63
Beta-blockers, *n* (%)	897 (95.3)	0.67 (0.24–1.84)	0.43	854 (95.4)	43 (93.5)	0.8
ACE-inhibitors, *n* (%)	885 (94)	0.56 (0.24–1.31)	0.18	842 (94.1)	43 (93.5)	1
Statins, *n* (%)	879 (93.4)	0.52 (0.24–1.14)	0.1	837 (93.5)	42 (91.3)	0.78
Diuretics, *n* (%)	415 (44.1)	2.11 (1.26–3.51)	0.004	388 (43.4)	27 (58.7)	0.06
GRACE score	110.2 (93.4–25.8)	1.04 (1.03–1.05)	<0.001	109.3 (92.5–125.4)	121.7 (109.9–136.0)	0.001
eGFR Counahan Barratt	71.9 (62.4–82.7)	0.97 (0.95–0.98)	<0.001	72.2 (62.4–83.1)	67.6 (54.7–75.4)	0.02

ACE, angiotensin-converting enzyme; BMI, body mass index; CABG, coronary artery bypass graft; CK, serum creatine kinase; COPD, chronic obstructive pulmonary disease; CPR, cardiopulmonary resuscitation; IQR, inter-quartile range; LVEF, left ventricular ejection fraction; MI, myocardial infarction; PCI, percutaneous coronary intervention. *Hazard ratio refers to risk of total mortality.

### Model-Based QTV Analysis Results

The average QT interval and rate-corrected QT interval were 425.7 ± 44.8 ms and 432.6 ± 39.7 ms, respectively. The variance of beat-to-beat fluctuations in QT interval was 10.4 ± 24.7 ms^2^ on average.

**FIGURE 1:**
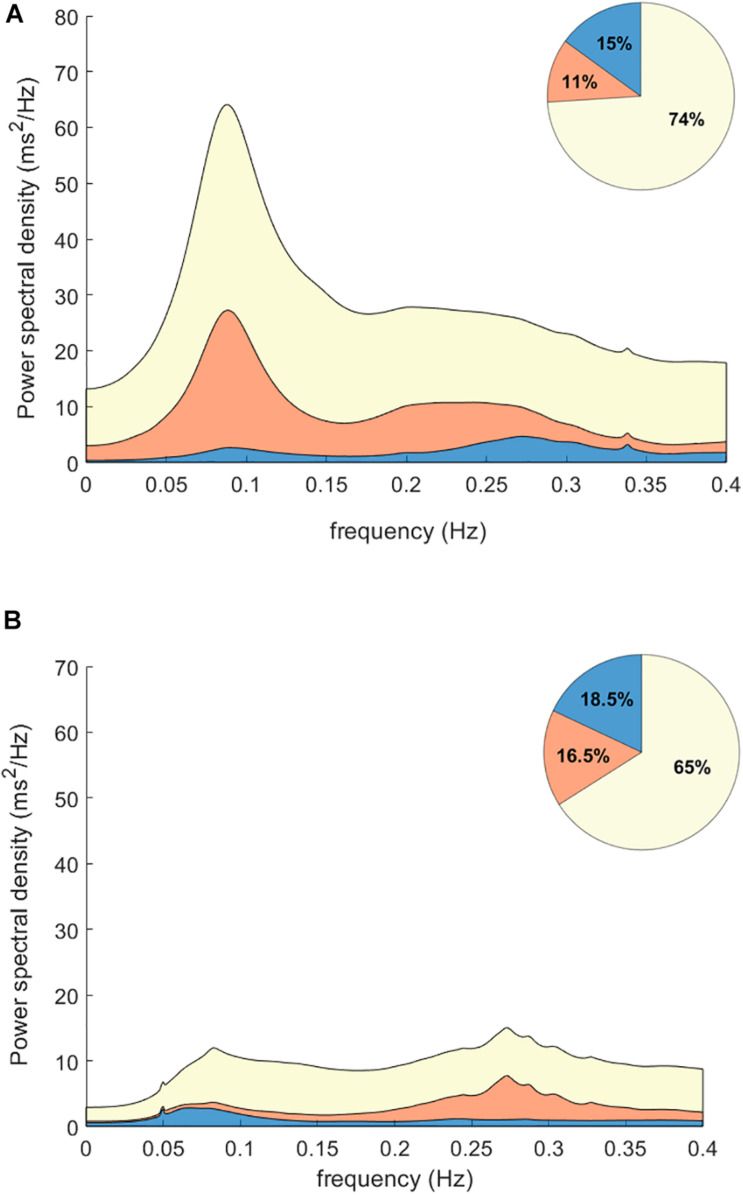
Power contributions to QT interval variability. Plots show the spectrograms averaged for non-survivors **(A)** and survivors **(B)** post MI. The relative contribution of RR interval variability is shown in blue; the contribution of respiration is shown in orange. While the absolute value of the RR (blue) and respiration (orange) contributions are higher for non-survivors due to the overall increase in QTV in that group, the relative contribution of RR and respiration to QT power is decreased in non-survivors as shown in the pie charts. Notably, the QT power independent of RR and respiration (yellow) is increased and exhibits a clear peak in the low-frequency band.

Figure 1 shows the relative contribution of RR and respiration to total QTV in survivors, and non-survivors averaged across all patients. While the absolute value of the RR and respiration contribution is higher for non-survivors due to the overall increase in QT power in that group, the relative contribution of RR and respiration to QTV is decreased in non-survivors as demonstrated by the pie charts. The QT power independent of RR and respiration is increased and exhibits a clear peak in the low-frequency band.

**FIGURE 2:**
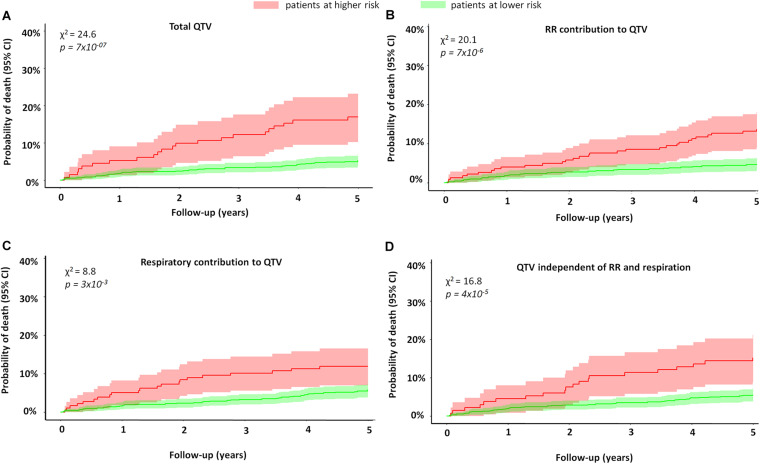
Mortality of MI patients stratified by QTV and its components. The figure shows the Kaplan–Meier curves of MI patients stratified by **(A)** total QT variability, **(B)** percentage of RR contribution to QTV, **(C)** percentage of respiratory contribution to QTV, and **(D)** percentage of QTV independent of RR and respiration. The red curve represents patients at higher risk, while the green curve represents those with lower risk. Patients with higher QTV had a higher risk of mortality compared to those with lower QTV (16.8% vs. 5.5%, *p* < 0.001). Patients with higher QTV independent of RR and respiration also had a higher 5-year mortality rate (15.2% vs. 5.5%, *p* < 0.001). Finally, the 5-year mortality rate was higher for patients with reduced RR and respiratory contributions to QTV (13.5% vs. 4.8%; 11% vs. 5.7%, respectively). The figure demonstrates the univariate predictive value of QTV and its components is risk stratification.

Figure 2 shows the Kaplan–Meier curves for QTV and its individual components. An increase in QTV and QTV independent of RR and respiration was associated with increased risk of mortality (QTV:16.8% vs. 5.5%, *p* < 0.001; QTV independent:15.2% vs. 5.5%, *p* < 0.001). On the other hand, the 5-year mortality rate was higher for patients with reduced RR and respiratory contributions to QTV (13.5% vs. 4.8%, *p* < 0.001; 11% vs. 5.7%, *p* = 0.003, respectively).

### Cox Regression Analysis

In univariate Cox regression analysis (Table 2), all QT variables were significantly associated with mortality except for QTV_respiration_. Multivariable stepwise Cox regression analysis of QT variables identified the increase in QTV_total_ and QTV_unexplained_ as significant predictors of mortality in addition to QT_c_ prolongation (Table 2). A linear predictor score (termed QTV risk score) was calculated from a linear combination of these significant predictors.

**TABLE 2:** Univariate and multivariable Cox regression analysis of QT variables.

Variable	Univariate		Multivariable (All variables in)	
	Hazard ratio (95%)	*p*-value	Hazard ratio (95%)	*p*-value
*QT*_c_ (ms)	1.01 (1.01–1.02)	<0.001	1.010 (1.01–1.03)	0.001
*QTV*_total_ (ms^2^)	1.01 (1.006–1.014)	<0.001	1.005 (1.00–1.01)	0.08
*QTV*_respiration_ (%)	0.99 (0.97–1.005)	0.168	-	-
*QTV*_RR_ (%)	0.96 (0.94–0.99)	0.004	-	-
*QTV*_unexplained_ (%)	1.03 (1.01–1.045)	0.001	1.024 (1.01–1.04)	0.004

Analysis of deviance demonstrated the added value of QTV_total_ and QTV_unexplained_ to the model when compared to a model with QT_*c*_ alone (χ^2^ = 12.2, *p* = 0.002). Using stepwise multivariable cox regression, we explored whether the QTV risk score adds predictive value to a model consisting of the following clinical predictor variables (model details can be found in Table 3): GRACE score, LVEF, presence of diabetes, chronic obstructive pulmonary disease and expiration-triggered respiratory sinus arrhythmia. Here, the QTV risk score added significant predictive value with an HR = 1.73 (1.23–2.51), *p* = 0.002 (Table 4).

**TABLE 3:** Multivariable Cox regression analysis of clinical risk markers.

Variable	Hazard ratio (±SE)	*p*-value
GRACE score	1.04 (±0.006)	<0.001
ETA	0.79 (±0.04)	<0.001
LVEF	0.97 (±0.01)	0.007
Diabetes	1.72 (±0.27)	0.047
COPD	2.19 (±0.39)	0.044

*GRACE, global registry of acute coronary event; ETA, expiration triggered arrhythmia; LVEE, left ventricular ejection fraction; COPD, chronic obstructive pulmonary disease.*

**TABLE 4:** Hazard ratios and 95% confidence intervals of the clinical and QTV models in the multivariable cox regression analysis.

Variables	Hazard ratio^a^ (95% CI)	*p*-value
Clinical model^b^	2.45(2.2–3.3)	<0.001
QTV risk score^c^	1.73 (1.23–2.51)	0.002

*^a^ Hazard ratio refers to the risk of total mortality. ^b^Model details in Table 3. ^c^Model details in Table 2.*

**FIGURE 3:**
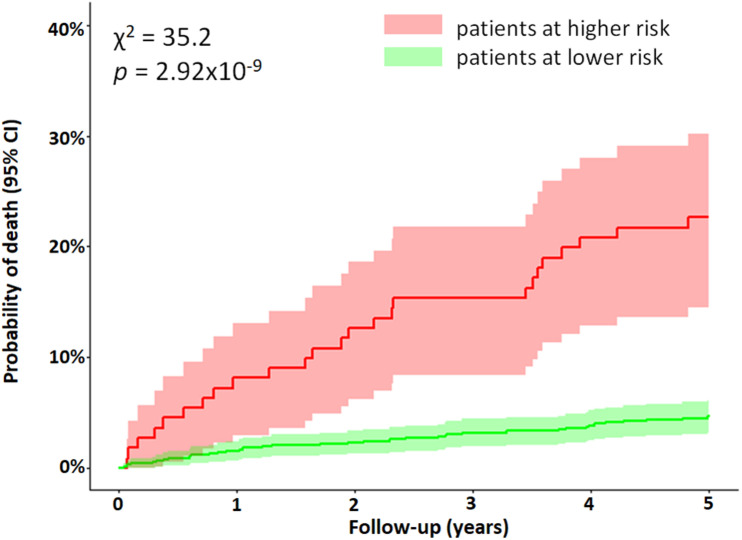
Mortality of MI patients stratified by the QTV risk score. Kaplan–Meier curves were obtained from the dichotomized multivariable Cox regression model. The red curve represents patients above the cut off value (0.626), while the green curve represents all other patients. Difference between the two curves was tested using the log-rank test (χ^2^ = 35.2, *p* = 2.92×10^– 9^). The figure demonstrates the predictive value of an increase in QTV risk score for identifying patients with a higher risk of mortality post MI.

Kaplan–Meier curves of the dichotomized QTV risk score are shown in Figure 3. The 5-year mortality rate for patients with a high QTV risk score was significantly higher compared to those with a lower QTV risk score (18.4% vs. 4.7%, *p* = 2.92×10^–9^).

**FIGURE 4:**
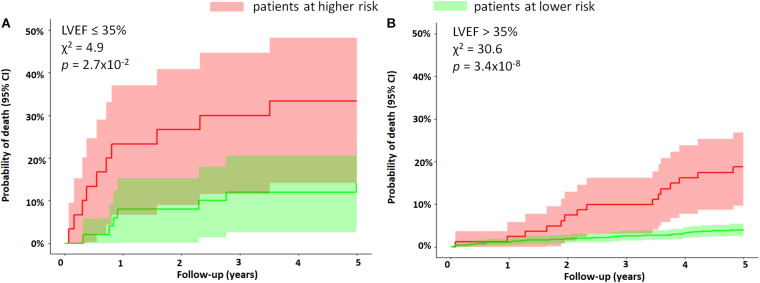
Subgroup analysis based on LVEF. The figure shows the Kaplan–Meier curves of MI patients stratified by the QTV risk score for subgroups with **(A)** LVEF ≤ 35% and **(B)** LVEF > 35%. The 5-year mortality rate was significantly higher in patients with LVEF > 35% who had a high QTV risk score (16% vs. 4%, *p* = 1.19×10^– 7^). The QTV risk score was only marginally predictive of mortality in patients with LVEF ≤ 35%. The figure demonstrates the ability of the QTV risk score to further stratify a subgroup at high risk in the group with LVEF > 35%.

The cohort was divided into subgroups based on LVEF. In the group with LVEF > 35%, the 5-year mortality risk was significantly higher for patients who had a high QTV risk score (16% vs. 4%, *p* = 1.19×10^–7^). The QTV risk score was also marginally predictive of mortality in patients with LVEF ≤ 35% (Figure 4).

**FIGURE 5:**
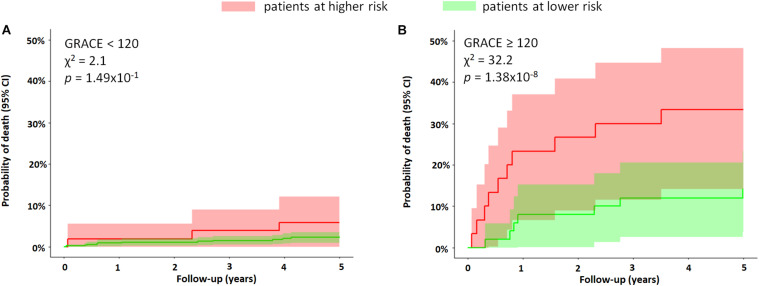
Subgroup analysis based on GRACE score. The figure shows the Kaplan–Meier curves of MI patients stratified by the QTV risk score for subgroups with **(A)** GRACE score < 120 and **(B)** GRACE score ≥ 120. The QTV risk score was significantly predictive of mortality only in patients with a score ≥ 120, where the 5-year mortality rate was 25% versus 11%, *p* = 0.0009. The figure demonstrates the ability of the QTV risk score to further stratify a subgroup with a higher risk in the high-risk cohort (GRACE ≥ 120).

In a subgroup of patients with GRACE score ≥ 120, patients with a higher QTV risk score had a significantly higher 5-year mortality risk compared to those with lower QTV risk score in the same group (25% vs. 11%, *p* = 0.0009) (Figure 5).

## Discussion

Our study demonstrates that heart rate-independent mechanisms play a dominant role in creating excessive QTV observed in MI patients with increased mortality risk. The novel QTV risk score yields improved risk stratification within a subgroup of MI patients who already display traditional risk markers (GRACE score ≥ 120). It can also identify vulnerable patients in patient groups with normal to moderate LVEF (LVEF > 35%).

Repolarization lability can trigger malignant arrhythmias and subsequent SCD in patients post MI (Liew, 2010). QTV was found to be elevated during ischemic episodes compared to non-ischemic episodes (Murabayashi et al., 2002). We found that QTV is elevated in non-surviving MI patients when compared to those who survived.

Previously, simple QTV metrics were combined with conventional risk markers to identify MI patients at high risk of mortality (Jensen et al., 2005; Piccirillo et al., 2007). Here, we sought to decompose QTV into its physiological sources and assessed their individual role in risk stratification. We found that the relative contribution of RR interval variability to QTV was reduced in non-survivors, indicating attenuated rate-adaptation of the QT interval. This attenuation of the QT-RR relationship has also been found in MI patients with reduced LVEF when compared to those with coronary artery disease but no MI (Sosnowski et al., 2002). Others found no difference in the RR dependent component of QTV when comparing MI patients to healthy subjects (Lombardi et al., 1998; Zhu et al., 2008). These aggregate results suggest that the attenuation in QT-RR relationship is only prevalent in high-risk patients rather than in all patients post MI. Possibly, the attenuation is linked to increased sympathetic activation (Zaza et al., 1991; Porta et al., 2010; El-Hamad et al., 2015). Holter recordings showed substantial predictive value of attenuated rate-adaptation of the QT interval for SCD in MI patients (Hintze et al., 2002; Chevalier et al., 2003).

In our study, the respiratory contribution to QTV did not play a significant role in predicting patients at risk in the univariate model (Table 2). Hence, we considered a simplified model without respiration. It’s predictive performance was comparable to the original model (data not shown), suggesting that recording of respiration is not necessary. Contrary to our findings, others have reported an increase in high frequency of RT interval variability in patients with reduced LVEF compared to those with preserved LVEF or with coronary artery disease but no MI (Sosnowski et al., 2002).

The portion of QTV that cannot be explained by rate-adaptation or respiration-related mechanisms was increased in patients who died during the follow-up period (Table 2). In healthy subjects, the unexplained fraction of QTV has been previously shown to correlate with an increase in sympathetic tone during tilt (Porta et al., 2010; El-Hamad et al., 2015) and is augmented in post-MI patients compared to healthy subjects (Zhu et al., 2008).

The pathophysiological mechanisms that increase overall QTV and QTV independent of heart period and respiration and contribute to mortality risk are not fully understood. By design, our study does not facilitate the direct interpretation of the QTV component independent of heart rate and respiration. However, the spectral component of QTV that is independent of RR and respiration exhibits a clear peak in the low-frequency (LF; 0.04-0.15 Hz) region that is elevated in the non-survivors, as shown in Figure 1. It is well-known that sympathetic nerve activity oscillates at the LF rhythm, creating Traube–Hering–Mayer waves in arterial blood pressure that coincide with oscillations in RR variability. Sympathetic nerve activity modulates the relationship between repolarization and cycle length in the LF band (Zaza et al., 1991). It also contributes directly to the magnitude of QTV by affecting L-type channel Ca^2+^ current and the slow delayed rectifier potassium current in ventricular myocytes. Nerve sprouting (Zhou et al., 2004) and sympathetic hyperinnervation following acute MI can create electrical instability, where augmented sympathetic activity can lead to malignant ventricular arrhythmias (Cao et al., 2000). Other studies suggest that systemic inflammation post-MI could contribute at least in part to the increase in QTV (Tseng et al., 2012). Since the severity and prognosis of myocardial infarction are highly dependent on the size and location of the infarcted tissue, affecting the extent of spatial repolarization heterogeneity (Hiromoto et al., 2006), QTV independent of heart rate and respiration may also be affected by the characteristics of the damaged substrate.

Markers of an abnormal substrate or structural heart disease are some of the most common risk factors for SCD, and reduced LVEF is currently considered the most important marker for risk stratification (Wellens et al., 2014). Current international guidelines for implantable cardioverter defibrillator implantation for primary prevention are predominantly based on LVEF (O’Gara et al., 2013; Priori et al., 2015). However, the majority of SCDs occur in patients with preserved LVEF (LVEF > 30%) (Buxton et al., 2007; Wellens et al., 2014). Therefore, the identification of high-risk patients in this cohort is critical.

Several markers of electrical instability and autonomic tone, such as heart rate variability, heart rate turbulence, T wave alternans, and QTV index, have been associated with increased risk of SCD in post MI patients with preserved LVEF (Piccirillo et al., 2007; Gatzoulis et al., 2019). In this group of patients, we found that a higher QTV risk score is associated with a four times higher risk of mortality compared to those with lower values of QTV risk score. Gatzoulis et al. (2019) reported that patients with preserved LVEF who display at least one ECG-derived non-invasive risk factor were at higher risk of SCD. As QTV reflects lability of the ventricular repolarization process, a higher QTV risk score could identify patients more susceptible to ventricular arrhythmia.

The QTV risk score was only marginally predictive in the group with reduced LVEF. This could be explained by the fact that patients with reduced LVEF have a higher competing risk of death from heart failure than other modes of death when compared to patients with preserved LVEF (Hall et al., 2018). The GRACE score is a hospital discharge risk score shown to be predictive of mortality in patients with acute coronary syndrome. It combines multiple prognostic factors such as age, history of heart failure and MI, and heart rate (Tang et al., 2007). Patients with a GRACE score ≥ 120 are considered to be at high risk. In our study, the QTV risk score was able to identify a subgroup of patients at higher risk within the subgroup of high-risk patients identified by a GRACE score ≥ 120.

Our study has several limitations. Current guidelines recommend using 12-lead ECG screening for specific high-risk patients (Priori et al., 2015); we used single-lead ECG in favor of lower complexity and cost. By choosing the lead with the largest T wave and the lowest noise, we obtained a reasonably good approximation of the global repolarization duration. However, potentially valuable information on the repolarization dispersion across leads could not be assessed. Although we used high-resolution ECG, conventional ECG sampled at > 300 Hz is sufficient for QTV analysis (Baumert et al., 2016a). Patients were studied at rest, and while autonomous nervous system provocation tests tend to increase the predictive power of ECG markers, their practical use in patients is limited due to the complexity of tests (Wellens et al., 2014). Stationary segments of heart period, QT interval and respiratory time series were present in all recordings as judged by visual assessment. Fully automated methods should be considered in the future for a more rigorous assessment. The 5-year mortality rate in our cohort was low, and hence, the identified groups with increased mortality may be small. This may have impacted our analysis by possibly reducing its statistical power and broadening the confidence intervals of the Kaplan–Meier curves, as seen in Figures 3–5. Accurate identification of specific patients at risk based on QTV alone might be challenging. Patients who were not in sinus rhythm were excluded from the analysis, which introduced a mortality bias. We did not systematically measure serum creatine kinase-muscle/brain and troponin levels. Since the study enrollment, improved treatments for MI have become clinically available (e.g., improved stents and antithrombotic drugs) that may have affected our findings. Our follow-up protocol did not include data collection on change in patient characteristics and therapy adherence. The primary study endpoint was all-cause mortality. Future studies should validate our findings on data from a different cohort stratifying for cardiovascular mortality, in particular, SCD.

## Conclusion

In conclusion, the QTV risk score might help stratify high-risk patients that already display traditional risk markers and identify patients at higher risk of mortality, albeit their preserved LVEF. The implementation of risk stratification strategies to predict risk in patients with preserved LVEF requires the use of a combination of several risk markers. New stratification strategies which can be translated into daily clinical practice are necessary to stratify patients with moderate and normal LVEF yet are at higher risk of SCD. The QTV risk score is a non-invasive risk marker that can be easily incorporated into daily clinical practice. However, the prognostic value of the QTV risk score needs to be prospectively validated on other cohorts, and further investigation into its utility for specifically predicting SCD is required.

## Data Availability Statement

The data presented in this article are not readily available. Data access requests should be emailed to author Georg Schmidt directly.

## Ethics Statement

The studies involving human participants were reviewed and approved by the ethics committees at the hospital of the Technische Universität München, the German Heart Centre, and the Klinikum Rechts der Isar, both in Munich, Germany. The patients/participants provided their written informed consent to participate in this study.

## Author Contributions

FE-H was responsible for the conception, modeling, analysis, and interpretation of data; for drafting of the manuscript and revising it critically for important intellectual content; and for the final approval of the manuscript submitted. SB was responsible for the processing and analysis of data; for revising the manuscript critically for important intellectual content; and for the final approval of the manuscript submitted. AM was responsible for the analysis and interpretation of data; for drafting of the manuscript; and for the final approval of the manuscript submitted. AS was responsible for the conception, design, and analysis of the data; for revising the manuscript critically for important intellectual content; and for the final approval of the manuscript submitted. GS was responsible for the conception, design, analysis, and interpretation of data; for revising the manuscript critically for important intellectual content; and for the final approval of the manuscript submitted. MB was responsible for the conception, design, and interpretation of data; for drafting of the manuscript and revising it critically for important intellectual content; and for the final approval of the manuscript submitted. All authors contributed to the article and approved the submitted version.

## Conflict of Interest

The authors declare that the research was conducted in the absence of any commercial or financial relationships that could be construed as a potential conflict of interest.
